# Dynamics of Docosahexaenoic Acid Utilization by Mouse Peritoneal Macrophages

**DOI:** 10.3390/biom13111635

**Published:** 2023-11-10

**Authors:** Patricia Monge, Alma M. Astudillo, Laura Pereira, María A. Balboa, Jesús Balsinde

**Affiliations:** 1 Instituto de Biología y Genética Molecular, Consejo Superior de Investigaciones Científicas (CSIC), 47003 Valladolid, Spaina.astudillo@uva.es (A.M.A.); mbalboa@uva.es (M.A.B.); 2 Centro de Investigación Biomédica en Red de Diabetes y Enfermedades Metabólicas Asociadas (CIBERDEM), Instituto de Salud Carlos III, 28029 Madrid, Spain

**Keywords:** docosahexaenoic acid, arachidonic acid, lipid signaling, membrane phospholipid, inflammation, monocytes/macrophages

## Abstract

In this work, the incorporation of docosahexaenoic acid (DHA) in mouse resident peritoneal macrophages and its redistribution within the various phospholipid classes were investigated. Choline glycerophospholipids (PC) behaved as the major initial acceptors of DHA. Prolonged incubation with the fatty acid resulted in the transfer of DHA from PC to ethanolamine glycerophospholipids (PE), reflecting phospholipid remodeling. This process resulted in the cells containing similar amounts of DHA in PC and PE in the resting state. Mass spectrometry-based lipidomic analyses of phospholipid molecular species indicated a marked abundance of DHA in ether phospholipids. Stimulation of the macrophages with yeast-derived zymosan resulted in significant decreases in the levels of all DHA-containing PC and PI species; however, no PE or PS molecular species were found to decrease. In contrast, the levels of an unusual DHA-containing species, namely PI(20:4/22:6), which was barely present in resting cells, were found to markedly increase under zymosan stimulation. The levels of this phospholipid also significantly increased when the calcium-ionophore A23187 or platelet-activating factor were used instead of zymosan to stimulate the macrophages. The study of the route involved in the synthesis of PI(20:4/22:6) suggested that this species is produced through deacylation/reacylation reactions. These results define the increases in PI(20:4/22:6) as a novel lipid metabolic marker of mouse macrophage activation, and provide novel information to understand the regulation of phospholipid fatty acid turnover in activated macrophages.

## 1. Introduction

Macrophages are highly specialized phagocytic cells that participate in a diverse array of innate immune responses and inflammatory processes [1,2,3,4,5]. Their pivotal role in orchestrating immunoinflammatory reactions has been attributed to a significant extent to their ability to generate various chemical signalers and autacoids. Among these bioactive compounds are the oxygenated derivatives of arachidonic acid (AA), collectively referred to as the eicosanoids [6,7,8]. This group of compounds includes the prostaglandins, thromboxane, leukotrienes, hydroxyeicosatetraenoic acids, and lipoxins [9].

Importantly, AA is not the sole polyunsaturated fatty acid subject to conversion into potent bioactive mediators during inflammatory processes. Docosahexaenoic acid (DHA), the predominant n-3 fatty acid in innate immune cells [10], has also been shown to undergo transformation into potent mediators through various enzymatic pathways, including the sequential involvement of 5-, 12-, and/or 15-lipoxygenases [11]. Depending on the specific lipoxygenase form responsible for catalyzing the reaction, DHA-derived metabolites can be categorized into three main types: resolvins, protectins, and maresins [12]. While the pathways of formation of DHA-derived metabolites and some of their biological actions are yet to be conclusively established [13,14], each type of DHA metabolite is believed to exert complementary pro-resolving effects and interact with distinct receptors [15,16]. As a result, they play a role in regulating diverse cell types and signaling pathways. In all, these discoveries highlight the capacity of both n-6 and n-3 fatty acids to serve as precursors for potent bioactive molecules with multifaceted functions in innate immunity and inflammation.

It is widely recognized that the availability of free AA is a critical factor governing the rate of cellular eicosanoid synthesis [17,18,19]. AA serves as an intermediate in a deacylation/reacylation process involving membrane glycerophospholipids, a process often referred to as the Lands cycle [20,21]. In this cycle, the fatty acid is cleaved from the sn-2 position of glycerophospholipids through the action of phospholipase A_2_s (PLA_2_) and subsequently reintegrated into phospholipids via CoA-dependent acyltransferases. The enzymes participating in the Lands cycle, in conjunction with CoA-independent transacylases responsible for transferring AA moieties among various phospholipid classes, collectively contribute to the uneven distribution of AA in cellular phospholipids [22,23,24,25,26,27]. A striking aspect of this distribution is the marked enrichment of some phospholipid species as opposed to others. For instance, in innate immune cells, the ethanolamine plasmalogens are markedly enriched with AA, suggesting pivotal roles for these particular species in maintaining cellular AA homeostasis [27,28,29,30,31]. This non-uniform distribution of AA within cells also plays a role in eicosanoid regulation, as it appears to dictate the preferential production of certain eicosanoids based on the original phospholipid source of AA [31,32,33,34,35,36,37]. Thus, the distribution of AA among distinct cellular compartments may represent a crucial limiting step for the synthesis of eicosanoids [27].

The mechanisms governing the cellular utilization of DHA and other n-3 polyunsaturated fatty acids have received relatively less attention. By analogy with AA, it is frequently assumed that the same principles governing AA dynamics are also applicable to DHA. While this assumption may hold true in a broad sense, there may exist notable differences. For instance, cytosolic group IVA PLA_2_ (cPLA_2_α) is by far the major enzyme effecting the AA release from stimulated cells [38,39,40,41,42]. However, studies with cells from mice with genetic deletion of another PLA_2_, i.e., the group VIA calcium-independent enzyme (iPLA_2_β), have established the key role of iPLA_2_β, but not of cPLA_2_α, in regulating the mobilization of DHA from phospholipids [43,44]. This illustrates the complexities and interrelations of phospholipid fatty acid metabolism and utilization by innate immune cells.

Using mass spectrometry-based lipidomic approaches, we report in this study the characterization of the molecular species of glycerophospholipids involved in DHA homeostasis in resting and activated mouse macrophages. Our study also identifies a DHA-containing phospholipid in stimulated cells that exhibits a negligible presence in resting cells. This is, to the best of our knowledge, the first work reporting the various molecular species that contribute to the mobilization of DHA from phospholipids and the generation of novel species that may help to define specific macrophage activation states.

## 2. Materials and Methods

### 2.1. Cell Culture

Resident peritoneal macrophages from Swiss male mice (University of Valladolid Animal House, 10–12 weeks old) were obtained by peritoneal lavage as described elsewhere [45]. All procedures involving animals were undertaken under the supervision of the Institutional Committee of Animal Care and Usage of the University of Valladolid (Approval No. 7406000), and are in accordance with the guidelines established by the Spanish Ministry of Agriculture, Food, and Environment and the European Union. RAW264.7 macrophage-like cells were cultured as previously described [46,47]. For experiments, the cells were placed in serum-free medium for 1 h before the addition of stimuli and/or inhibitors. The latter were routinely added 30 min before the former. Zymosan was prepared as described [48]. Batches that contained decided phospholipase A_2_ activity, as assessed by in vitro assay [49,50,51,52], were excluded. Protein content was assayed according to Bradford [53] using a commercial kit (BioRad Protein Assay, Bio-Rad, Hercules, CA, USA).

### 2.2. Measurement of DHA Incorporation into Phospholipids and Phospholipid Remodeling

The cells were exposed to exogenous [^14^C]DHA (sp. act. 55 mCi/mmol; American Radiolabeled Chemicals, St. Louis, MO, USA) (0.5 µM; 0.l µCi/mL) for different periods of time. Total lipids in the cell monolayers were extracted according to Bligh and Dyer [54]. Phospholipids were separated from neutral lipids by thin-layer chromatography, using n-hexane/diethyl ether/acetic acid (70:30:1, *v*/*v*/*v*) as a mobile phase [55]. For the separation of phospholipid classes, plates impregnated with boric acid were used [56], and the mobile phase consisted of chloroform/methanol/28% ammonia (65:25:5, *v*/*v*/*v*) [57,58]. The different bands were scraped from the plates and their radioactive content was determined by scintillation counting. Phospholipid fatty acid remodeling was carried out exactly as described by Lebrero et al. [31].

### 2.3. Mass Spectrometry Analyses of Phospholipids

The analysis of phospholipid molecular species was carried out by liquid chromatography coupled to mass spectrometry (LC-MS) exactly as described [59], using a Thermo Scientific Dionex Ultimate 3000 high-performance liquid chromatograph coupled online to a QTRAP 4500 mass spectrometer (AB Sciex, Framingham, MA, USA). Analyses of the fatty acid composition of the various phospholipid classes was carried out by gas chromatography coupled to mass spectrometry (GC-MS) exactly as described [60], using an Agilent 7890A gas chromatograph coupled to an Agilent 5975C mass-selective detector operated in electron impact mode (Agilent Technologies, Santa Clara, CA, USA).

### 2.4. Statistical Analysis

Data are described as means ± standard error of the mean. Statistical significance was determined by Student’s *t* test, and one-way ANOVA followed by Tukey’s post hoc test conducted in SigmaPlot software, version 14.0 (Systat Software Inc., San Jose, CA, USA).

## 3. Results

**Figure 1:**
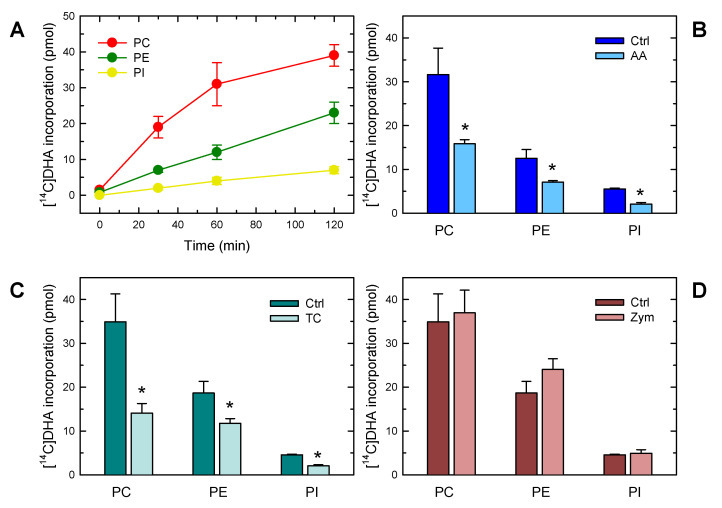
Characterization of DHA incorporation into macrophage phospholipids. (**A**) Time-course of incorporation into different phospholipid classes. The cells were incubated with [^14^C]DHA, and incorporation of the fatty acid was measured in PC (red), PE (green) and PI (yellow). (**B**) Effect of AA on the incorporation of [^14^C]DHA into different phospholipid classes. The cells were exposed to [^14^C]DHA for 60 min in the absence (blue) or presence (light blue) of AA. (**C**) Effect of triacsin C (TC) on the incorporation of [^14^C]DHA into different phospholipid classes. The cells were exposed to [^14^C]DHA for 60 min in the absence (green) or presence (light green) of 3 µM triacsin C. (**D**) Effect of zymosan stimulation on the incorporation of [^14^C]DHA into different phospholipid classes. The cells were exposed to [^14^C]DHA for 60 min in the absence (maroon) or presence (light maroon) of 0.5 mg/mL zymosan, which acted as a cellular stimulant. The results are shown as means ± standard error of the mean (*n* = 3). * *p* < 0.05, significance of control (Ctrl) cells versus cells treated with AA, TC, or zymosan.

Exposure of murine peritoneal macrophages to exogenous [^14^C]DHA resulted in the rapid incorporation of the fatty acid phospholipids (Figure 1A). Choline glycerophospholipids (PC) constituted the major reservoir for the fatty acid, followed by ethanolamine phospholipids (PE) and phosphatidylinositol (PI). It is worth noting that the rate of incorporation into PC and PI seemed to slow down after 1 h of exposure, while the incorporation into PE proceeded linearly for at least 2 h (Figure 1A). When the [^14^C]DHA incorporation experiments were carried out in the presence of equimolar amounts of AA, a strong inhibition of the response was observed. All phospholipid classes, namely PC, PE and PI, were affected (Figure 1B). These data suggest that phospholipid DHA incorporation utilizes the same enzyme effectors as those used for AA incorporation. Consistent with this notion, phospholipid [^14^C]DHA incorporation into all phospholipid classes was strongly blunted by triacsin C, an acyl-CoA synthetase inhibitor that is also well known to block phospholipid AA incorporation (Figure 1C) [61,62,63].

Yeast-derived zymosan has been utilized for many years as a model stimulus to investigate PLA_2_-dependent routes for fatty acid mobilization and lipid mediator production in murine macrophages [64,65,66,67,68,69,70]. Stimulation of the cells with zymosan did not significantly modify the extent of DHA incorporation into phospholipids (Figure 1D), suggesting that the activation state of the macrophage is not a key regulatory factor for the process to occur.

**Figure 2:**
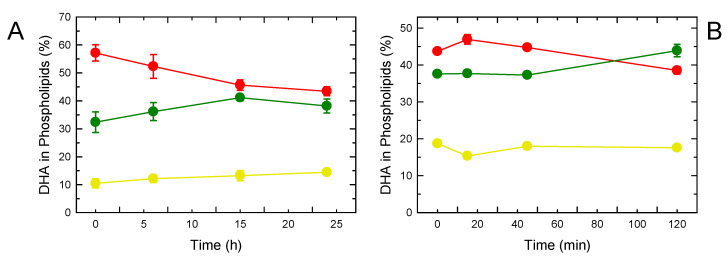
Phospholipid DHA remodeling. Mouse resident peritoneal macrophages (**A**) or RAW 264.7 macrophage-like cells (**B**) were pulse-labeled with [^14^C]DHA, washed, and incubated without label for the indicated periods of time. The radioactivity incorporated into each phospholipid class is given as a percentage of the radioactivity present in phospholipids. PC is indicated in red, PE in green, and PI in yellow. Data are shown as means ± standard error of the mean (*n* = 3).

Once incorporated into phospholipids, AA is known to be redistributed between the various phospholipid classes via direct transacylation reactions catalyzed by CoA-independent transacylase (CoA-IT). This enzyme transfers AA primarily from diacyl-PC to PE species, particularly the PE plasmalogens, without utilizing CoA or generating a free fatty acid intermediate [17,24,71]. These AA transacylation reactions are crucial to ensuring that AA is placed in the appropriate cellular compartments for the execution of specific cellular responses [26,27]. Therefore, it was important to analyze whether DHA is subject to a similar mechanism for the distribution of its levels among the various cellular phospholipid pools. To characterize this process, the macrophages were labeled with [^14^C]DHA for 30 min and the subsequent movement of the label between classes was measured (Figure 2). Consistent with the data shown in Figure 1A, immediately after the 30 min labeling period, PC was the major [^14^C]DHA-containing phospholipid, followed by PE and PI. Labeled DHA in PC experienced a sustained decrease with time, which was paralleled by a similar increase in PE, thus reflecting the action of CoA-IT (Figure 2A). These data demonstrate that DHA, like AA, experiences remodeling. The level of labeled DHA in PI did not change over time. To further characterize the phospholipid DHA remodeling, we also used RAW264.7 macrophage-like cells, which are known to remodel AA at a much faster rate than primary macrophages [31]. Figure 2B shows that DHA remodeling from PC to PE also occurred in the RAW264.7 cells at an expected faster rate. In turn, these data indicate that phospholipid DHA remodeling is a general mechanism operating in different macrophage cells.

In the next series of experiments, we turned to mass spectrometry-based analyses to characterize the specific molecular species where DHA is located in both resting and zymosan-stimulated macrophages. The goal of these studies was to identify the species that change during activation, and also new DHA-containing species that are synthesized as a consequence of cell stimulation. The latter may help to characterize novel signaling pathways of select macrophage activation states.

**Figure 3:**
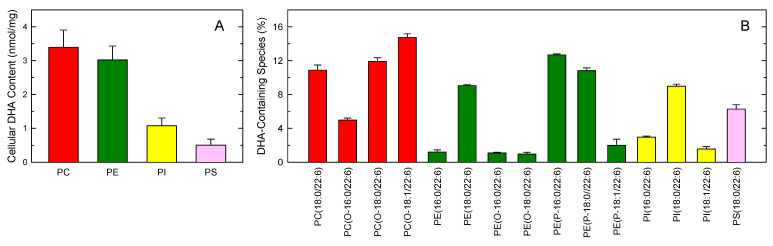
Phospholipid distribution of DHA in murine peritoneal macrophages. (**A**) Cellular DHA content in phospholipids, as assessed by GC-MS, is shown by class. (**B**) Profile of major DHA-containing phospholipid molecular species, as assessed by LC-MS. Choline phospholipids (PC) are shown in red; ethanolamine phospholipids (PE) are shown in green; phosphatidylinositol (PI) is shown in yellow; phosphatidylserine (PS) is shown in pink. The data are expressed as means ± standard error (*n* = 3).

Figure 3A shows the mass distribution of DHA between glycerophospholipid classes, as assessed by GC-MS. PC and PE contained similar amounts of DHA, with minor amounts being found in PI, and less so in PS. The total cellular DHA content was 7.99 ± 1.3 nmol/mg cell protein, which is consistent with previous estimates [10].

Figure 3B shows the distribution of DHA between the major phospholipid molecular species of the macrophages, as analyzed by liquid chromatography coupled to mass spectrometry (LC-MS). Following nomenclature recommendations [72,73], fatty chains within phospholipids are designated by their number of carbon atoms and number of double bonds, separated by a colon. A designation of O- before the first acyl chain indicates that the sn-1 position is ether-linked, whereas a P- designation indicates a plasmalogen form (sn-1 vinyl ether linkage). It is interesting to note the high levels of DHA present in ether phospholipids, i.e., the alkyl-PCs PC(O-18:0/22:6) and PC(O-18:1/22:6), and the ethanolamine plasmalogens PE(P-16:0/22:6) and PE(P-18:0/22:6). It is also remarkable that all four major DHA-containing diacyl-phospholipid species contain stearic acid (18:0) in the sn-1 position (i.e., PC(18:0/22:6), PE(18:0/22:6), PI(18:0/22:6), and PS(18:0/22:6), perhaps reflecting some sort of preference of the CoA-dependent acyltransferases using DHA as a donor for stearoyl-lysophospholipids among acyl-lysophospholipids.

**Figure 4:**
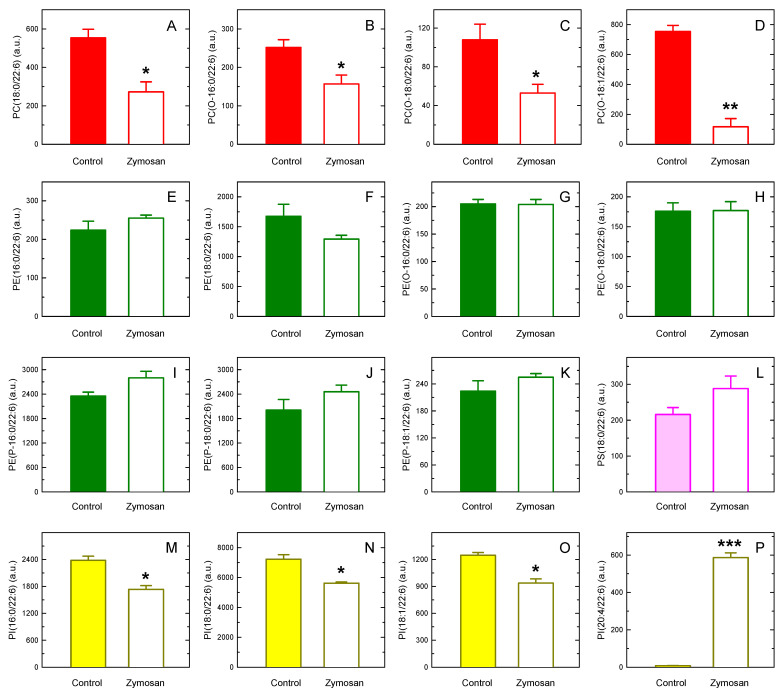
Changes in DHA-containing species after macrophage stimulation with zymosan. The cells were stimulated with 0.5 mg/mL zymosan for 1 h. Afterward, the content of DHA-containing PC (**A**–**D**), PE (**E**–**K**), PS (**L**), and PI (**M**–**P**) molecular species was determined via LC-MS. The data are shown as mean values ± standard error (*n* = 3). * *p* < 0.05, ** *p* < 0.01, *** *p* < 0.001, significantly different from the corresponding species in control unstimulated cells. a.u., arbitrary units.

Stimulation of the macrophages with zymosan for 1 h led to marked decreases in the DHA content of all molecular species of PC (Figure 4A–D) and PI (Figure 4M–O). DHA losses from PC species were generally stronger than those from PI species. Conversely, the DHA content of PE (Figure 4E–K) and PS (Figure 4L) molecular species was not significantly decreased. In fact, some of the species showed a tendency to increase their DHA content upon cellular activation. Given the data in Figure 2, showing that DHA undergoes remodeling from PC to PE species, we surmise that part of the DHA lost by PC species is directed to PE species, thereby attenuating or preventing the decline in DHA-containing PE species. Altogether, these results emphasize the differential roles played by the various phospholipid classes with regard to cellular DHA mobilization and utilization.

**Figure 5:**
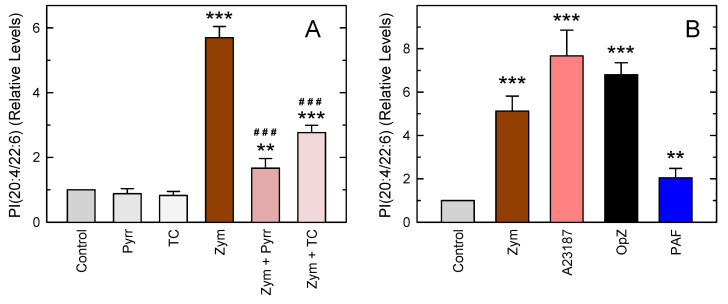
Characterization of PI(20:4/22:6) formation in mouse peritoneal macrophages. (**A**) Influence of pyrrophenone (1 µM, Pyrr) and triacsin C (3 µM, TC) on the levels of PI(20:4/22:6) after zymosan stimulation. (**B**) Effect of different stimuli on the production of PI(20:4/22:6). The cells were untreated (Ctrl) or treated with 0.5 mg/mL zymosan (Zym), 1 µM ionophore A23187, 0.5 mg/mL opsonized zymosan (OpZ), or 100 nM PAF for 1 h, as indicated. The data are shown as mean values ± standard error (*n* = 4). ** *p* < 0.01, *** *p* < 0.001, significantly different from control unstimulated cells. ^###^ *p* < 0.001, significantly different from zymosan-stimulated cells.

Importantly, a species identified as PI(20:4/22:6), which was barely detectable in resting cells, was readily observed in the zymosan-stimulated macrophages (Figure 4P). While cellular increases in the amounts of minor, but not unusual, phospholipids such as PC(20:4/20:4) and PI(20:4/20:4) have been described [28,74,75,76], the stimulated production of such an unusual phospholipid as PI(20:4/22:6) after cell activation is a novel and striking finding. Thus, we proceeded to characterize it further. Since no other phospholipid simultaneously containing AA (20:4) and DHA (22:6) was detected, it appears likely that PI(20:4/22:6) is formed via fatty acid exchange at both the sn-2 and sn-1 positions (i.e., the Lands pathway) [20,21], not via de novo using a phosphatidic acid intermediate already containing AA and DHA as lateral chains. In agreement with this assumption, Figure 5A shows that PI(20:4/22:6) production in activated cells was strongly inhibited when the cPLA_2_α inhibitor pyrrophenone [77,78,79] or the acyl-CoA synthetase inhibitor triacsin C [61,62,63] was present during the incubation.

To assess whether PI(20:4/22:6) formation was an event specific to cellular stimulation with zymosan, experiments were conducted with other widely used macrophage stimuli. Figure 5B shows that the calcium ionophore A23187 and opsonized zymosan were even more potent than zymosan in inducing PI(20:4/22:6) formation. On the other hand, platelet-activating factor (PAF) behaved as a weaker inducer. The finding that all tested stimuli induced PI(20:4/22:6) to varied degrees suggests that the formation of this phospholipid during stimulation may represent a general event of phospholipid fatty acid turnover in activated cells.

## 4. Discussion

By using radioactive labeling and mass spectrometry analyses, we describe in this work the dynamics of DHA utilization by mouse peritoneal macrophages with regard to phospholipid incorporation, distribution and release. The studies using radioactive fatty acid allowed us to demonstrate that DHA does not remain in the phospholipid classes where it is initially incorporated. Instead, a subsequent remodeling step occurs that distributes the fatty acid between various phospholipid pools. In this manner, while the major initial acceptor of DHA is PC, the amount of DHA in PE increases at the expense of PC after remodeling, eventually resulting in the presence of similar DHA levels in both PC and PE. These reactions appear to be similar to those previously described for AA [17,23]. Thus, the data in this study extend our knowledge on the phospholipid fatty acid remodeling pathways operating in macrophages to another polyunsaturated fatty acid, namely DHA. This has important pathophysiological implications, as the type and amounts of lipid mediators produced during activation likely depend on the composition and localization of the phospholipid pools used for the release of the precursor free fatty acid [26,27].

Our studies using mass spectrometry demonstrated that ether phospholipids are major endogenous reservoirs of DHA in murine peritoneal macrophages. It is remarkable in this regard that only one PC diacyl species, namely PC(18:0/22:4), ranks among the most abundant DHA-containing species of the macrophages. This contrasts with the distribution profile of AA within the same cells, where several other diacyl-PC species such as PC(16:0/20:4) or PC(18:1/20:4) are markedly enriched with AA [36]. Interestingly, analyses of the distribution of DHA between phospholipid species in cells from animals fed with DHA-enriched diets have also demonstrated the further enrichment of ether phospholipids with this fatty acid [80,81,82,83]. A similar enrichment in the ether phospholipid fraction was also found in studies with murine P388D_1_ macrophage-like cells cultured with DHA supplements [84]. Altogether, these results appear to indicate that the acyltransferases using DHA as an acyl donor manifest a pronounced affinity for lyso acceptors of the 1-alkyl or 1-alk-1′-enyl type [85].

Significant changes in the content of major DHA-containing phospholipid molecular species occur after stimulation of the macrophages with yeast-derived zymosan. The stimulated cells experience a marked loss in their DHA content in PC species and, to a lesser extent, in PI. Given that much of the DHA in PC species is present in ether-linked species, it appears reasonable to suggest that these species are prominent contributors to DHA mobilization in activated macrophages. This finding could be compatible with the possibility that the PLA_2_ effecting the stimulated DHA release exhibits some sort of selectivity for ether phospholipids. Notwithstanding this, a sharp decrease in the DHA levels of the major diacyl species PC(18:0/22:6), similar in qualitative terms to those of ether-linked PC species, was detected as well. Hence, the phospholipid composition of the intracellular location where the DHA-releasing PLA_2_ acts may also determine that the enzyme uses some substrates in preference over others. Whether any of the major intracellular PLA_2_s potentially capable of mediating fatty acid release from activated cells can distinguish the type of sn-1 linkage of the phospholipid substrate is currently under active investigation [27,86,87].

Another striking finding in the present study is that no net losses of DHA from PE are detected after macrophage stimulation. This could represent a situation similar to that described for AA [26,27,34,36]. Stimulation of the macrophages by a variety of agonists results in the activation of cPLA_2_α, which catalyzes the release of free AA from phospholipids [88,89,90,91,92]. In parallel, another enzyme, CoA-IT, directly transfers AA moieties from PC species to PE species. The latter include, prominently, the plasmalogens [93,94,95,96,97,98,99]. The final outcome of these phospholipid fatty acid remodeling reactions is that the AA mass levels in PE in activated cells are preserved. Given that our results show that DHA is also a substrate for transacylation reactions between phospholipids, a likely explanation for our findings is that the levels of DHA-containing PE in the activated cells are maintained, like those of AA, by the opposing balance between hydrolysis (mediated by PLA_2_), and reacylation (mediated by CoA-IT). In full agreement with our data, the deacylation of DHA from PE species would be followed by rapid transacylation of DHA moieties from PC. Ultimately, this would result in the replenishment of the DHA pool in PE at the expense of larger losses of DHA from PC. Moreover, the mild increases detected in the DHA content of several plasmalogen PE species after cell activation may indicate that the continuing action of CoA-IT exceeds that of PLA_2_ to ensure high levels of DHA in PE. This in turn suggests that DHA levels in PE might serve additional cellular roles in addition to participating in lipid mediator production.

In addition to PE, we also failed to detect changes in the DHA content of the species PS(18:0/22:6). To the best of our knowledge, we are not aware of PS being shown to act as an acceptor in the CoA-IT reaction [17,23]. Therefore, an alternative possibility is that the PLA_2_ responsible for effecting the release of DHA has no access to the cellular compartment where PS(18:0/22:6) is localized.

Interestingly, of all the DHA-containing phospholipid species measured, only one, namely PI(20:4/22:6), was found to increase its levels after macrophage activation with a variety of stimuli. The stimulated production of this species, which was barely detectable in resting cells, was strongly prevented by treating the cells with either the cPLA_2_α inhibitor pyrrophenone or the acyl-CoA synthetase inhibitor triacsin C. This pharmacological profile suggests that PI(20:4/22:6) is formed via deacylation/reacylation reactions driven by cPLA_2_α and CoA-dependent acyltransferases. Thus, in agreement with these data and also by analogy with the biosynthetic routes that have been dissected from studies adding exogenous fatty acids to the cells, it seems likely that PI(20:4/22:6) is formed not via de novo, but from a newly formed lysoPI acceptor. This is further discussed below.

As an authentic PI(20:4/22:6) standard is not available, at this time, we cannot unambiguously establish the regiospecific distribution of the two constituent fatty acids of the species. However, the production of PI(20:4/22:6) in activated macrophages is blocked by pyrrophenone, an exquisite inhibitor of cPLA_2_α-dependent AA hydrolysis. This suggests that DHA is most likely present at the sn-2 of the glycerol backbone, because the pyrrophenone data indicate that a PI species containing AA at the sn-2 position has to be the precursor of PI(20:4/22:6). Whether AA enters at the sn-1 position before or after the species has incorporated DHA at the sn-2 position cannot be answered at present. It is worth noting, however, that our previous studies assessing the pathway of formation of AA-containing PI species established that fatty acid recycling at the sn-1 position generally occurs when fatty acid recycling at the sn-2 position has already taken place [28,36]. This would indicate that AA is incorporated at the sn-1 position of PI(20:4/22:6) after DHA has been incorporated at the sn-2 position. Based on these considerations, we propose that, in activated cells, PI(20:4/22:6) is synthesized via a sequential mechanism whereby a PI species that contains AA at the sn-2 position is acted upon by cPLA_2_α to generate a 2-lysoPI which will be reacylated with DHA. Afterward, recycling with AA at the sn-1 position will occur, giving rise to the formation of PI(20:4/22:6). Of note, the existence of a pyrrophenone-sensitive step makes it highly unlikely that recycling of the sn-1 position of a pre-existing DHA-containing PI species with AA contributes significantly to the stimulated synthesis of PI(20:4/22:6).

The elevated levels of PI(20:4/22:6) that are found in activated cells may make this species a bona fide marker of macrophage responses to innate stimuli. Future studies should aim to investigate whether PI(20:4/22:6) possesses biological activity on its own, and thus it can be added to the growing number of defined phospholipid species with active roles in cell signaling [100,101,102,103,104,105]. The finding that PI(20:4/22:6) increases in response to a variety of proinflammatory stimuli of the macrophages suggests that it may be involved, directly or indirectly, in mediating inflammatory reactions. Thus, it may be relevant to those diseases with a marked inflammatory component where the exacerbation of macrophage lipid turnover and metabolism plays a key role, i.e., immunometabolic disorders [106].

## 5. Conclusions

Our study has revealed that, similar to AA, DHA is the subject of transacylation reactions that modify the initial distribution of this fatty acid between phospholipids. Hence phospholipid DHA remodeling contributes to shaping the equilibrium distribution of the fatty acid among the various phospholipid pools. Moreover, by using mass spectrometry-based lipidomic analyses, we describe that a DHA-containing species, PI(20:4/22:4), that is detected at very low levels under resting conditions, significantly increases in activated cells via a deacylation/reacylation pathway. Thus, this species could be regarded as a lipid marker of macrophage activation.

## Data Availability

Data are contained within the article.

## Abbreviations

AA, arachidonic acid (20:4n-6); DHA, docosahexaenoic acid (22:6n-3); CoA-IT, coenzyme A-independent transacylase; PC, choline-containing glycerophospholipids; PE, ethanolamine-containing glycerophospholipids; PI, phosphatidylinositol; PS, phosphatidylserine; PLA_2_, phospholipase A_2_, cPLA_2_α, group IVA cytosolic phospholipase A_2_α.
